# Imaging and Multidisciplinary Management of Acute Cholecystitis With Choledocholithiasis in a Phrygian Cap Gallbladder: A Case Report

**DOI:** 10.7759/cureus.81080

**Published:** 2025-03-24

**Authors:** Sophie Schuelke, Ambika Kapil, Odalys Frontela, Sohair Angly, Sahar S Abdelmoneim

**Affiliations:** 1 Medicine, Dr. Kiran C. Patel College of Osteopathic Medicine, Nova Southeastern University, Fort Lauderdale, USA; 2 Osteopathic Medicine, Dr. Kiran C. Patel College of Osteopathic Medicine, Nova Southeastern University, Davie, USA; 3 Internal Medicine, Palm Springs Campus, Larkin Community Hospital, Miami, USA; 4 Internal Medicine, Larkin Community Hospital, Miami, USA; 5 General Internal Medicine, Larkin Community Hospital, Hialeah, USA; 6 General Internal Medicine/Cardiovascular Medicine, Assiut University Hospital, Assiut, EGY

**Keywords:** acute cholecystitis, choledocholithiasis, ercp, hida cholescintigraphy, mrcp, phrygian cap

## Abstract

Anatomical variations in the gallbladder, such as a Phrygian cap, can complicate the diagnosis of acute cholecystitis, particularly when associated with choledocholithiasis. We report the case of a 43-year-old female who presented with complaints of right upper quadrant (RUQ) pain, which began one week prior and worsened over two days. The pain was associated with fever, nausea, and two episodes of vomiting the previous night. On admission, blood pressure was 104/66 mmHg, heart rate 61 bpm, respirations 18 bpm, saturating at 96%, pain 6, afebrile. The physical exam showed remarkable RUQ tenderness. Labs on admission were unremarkable apart from abnormal liver function testing (mild elevation of alkaline phosphatase (ALKP) 171 IU/L, alanine aminotransferase (ALT) 72 IU/L, and aspartate aminotransferase (AST) 48 IU/L. Ultrasound showed common bile duct dilation of approximately 11 mm without evidence of radiopaque choledocholithiasis. Sagittal computed tomography (CT) showed a distended and folded gallbladder, illustrating the anatomical variation of the Phrygian cap. Magnetic resonance cholangiopancreatography (MRCP) showed features suggestive of acute calculous cholecystitis and common bile duct filling defect consistent with choledocholithiasis. Hepatobiliary iminodiacetic acid (HIDA) scintigraphy provided evidence of acute cholecystitis and cystic duct obstruction. The patient was successfully treated with endoscopic retrograde cholangiopancreatography (ERCP), followed by a same-day laparoscopic cholecystectomy. Following three days of observation, the patient showed normal vital signs with decreasing epigastric pain. Additionally, lipase and amylase levels decreased from 366 and 1,440 U/L, respectively, to 176 and 100 U/L following ERCP. The patient was discharged home on Day 7 with acetaminophen, omeprazole, and instructions to follow up as an outpatient with both her primary and general surgery within 14 days. This case underscores the critical importance of a stepwise and multidisciplinary approach in the management of a complex case of acute cholecystitis.

## Introduction

Acute cholecystitis, often caused by gallstone obstruction of the cystic duct, is a common and potentially life-threatening condition. Anatomical variations, such as a Phrygian cap, can further complicate diagnosis and management, as demonstrated in this case [1]. It is one of the leading causes of abdominal pain, and if untreated, acute cholecystitis can progress to more severe complications, including gangrene, perforation, or the development of cholangitis and pancreatitis [2].

Gallbladder folds occur when the gallbladder wall folds onto itself, often affecting the posterior wall, and may result in unusual shapes like sigmoid or *boomerang* configurations [3]. Hartman’s pouch is defined as a fold near the neck of the gallbladder and is a common site of gallstone collection [4]. 

A Phrygian cap, the most common congenital gallbladder anomaly, involves folding of the fundus and can mimic pathological conditions on imaging, complicating diagnosis. This anomaly occurs as the result of the folding of the fundus during embryological development [1]. The folding of the gallbladder itself is not pathologic and is typically asymptomatic. This anatomic variation can however complicate the diagnosis of a symptomatic patient, such as in the case presented. Imaging can be difficult, as the Phrygian cap can result in shadowing or artifact, this may obscure gallstones or polyps, as well as mimic gallstones, sludge, mass, polyps, or obstruction leading to potential misdiagnosis [1,5]. Additionally, it might manifest as wall thickening, a crucial sign of acute cholecystitis, which could result in false-positive results [1,5]. Therefore, in the presence of symptoms concerning acute cholecystitis, such as nausea, vomiting, and/or localized RUQ or diffuse epigastric pain, further imaging with multiphase computed tomography (CT) or magnetic resonance imaging (MRI) is crucial in ruling out obstructions and masses [2]. Moreover, CT and MRI are better for anatomically viewing the gallbladder with a higher sensitivity and specificity for acute cholecystitis [1,5]. Cholescintigraphy, also known as a hepatobiliary iminodiacetic acid (HIDA) scan, may also be warranted in cases in which multiphase CT or MRI is inconclusive [6]. Sequential imaging is crucial to determining the correct diagnosis and underlying cause of acute cholecystitis. 

Herein we present the case of a patient with a Phrygian cap gallbladder with acute cholecystitis complicated by choledocholithiasis. Early diagnosis and prompt treatment are critical to avoiding complications. The anatomic variation of this patient’s gallbladder adds to the complexity of this case, as standard imaging may not be sufficient. In this case, a stepwise approach to imaging was implemented, which resulted in timely intervention with a combined approach involving ERCP and laparoscopic cholecystectomy. This case highlights the importance of prompt intervention in the management of this common and potentially dangerous condition.

## Case presentation

A 43-year-old female with a past medical history of gastritis presented to the emergency room with complaints of right upper quadrant (RUQ) abdominal pain, which began one week prior and worsened over two days. She described the pain as constant, non-radiating, and exacerbated with eating. She stated pain was associated with nausea and two episodes of vomiting the previous night. The patient was also febrile for one day before admission. 

On admission, the patient's vitals were stable: blood pressure 104/66 mmHg, heart rate 61 bpm, respirations 18 bpm, oxygen saturation 96%, and pain score 0. She was afebrile. The physical exam was unremarkable except for RUQ tenderness. Labs were unremarkable apart from mild elevations in alkaline phosphatase (ALKP) 171 IU/L, alanine transaminase (ALT) 72 IU/L, and aspartate transaminase (AST) 48 IU/L (Table 1). 

**Table 1:** Laboratory workup during the hospital course.

	Admission	Day 1	Day 2	Day 3	Day 4	Day 5	Day 6	Discharge (Day 7)	Reference range
Sodium (Na)	140	140	140	141	141	141	138	138	135-145 mmol/L
Potassium (K)	4.1	3.5	4.1	3.7	3.6	4.4	3.6	4.1	3.5-5.0 mmol/L
Chloride (Cl)	104	105	106	106	104	106	104	101	98-107 mmol/L
Bicarbonate (HCO3)	28	26	24	24	27	22	25	25	22-28 mmol/L
Blood urea nitrogen (BUN)	7	6	6	6	6	7	6	8	7-20 mg/dL
Creatinine	0.78	0.76	0.7	0.7	0.77	0.68	0.68	0.69	0.6-1.2 mg/dL
Calcium (Ca)	8.8	8.5	8.7	8.7	8.9	8.5	8.7	9	8.5-10.5 mg/dL
Total protein	8.1	7.6	7.6	7.5	7.8	7.4	7.4	7.1	6.0-8.3 g/dL
Albumin	4.2	3.8	3.8	3.8	4.0	3.9	3.8	3.8	3.5-5.0 g/dL
Globulin	3.9	3.8	3.7	3.7	3.8	3.5	3.6	3.3	2-3.5 g/dL
Amylase	N/A	N/A	N/A	N/A	N/A	1440	142	100	30-110 U/L
Lipase	N/A	N/A	N/A	N/A	N/A	366	373	176	10-140 U/L
Alkaline phosphatase	171	172	173	173	161	247	289	278	44-147 U/L
Alanine transaminase (ALT)	72	77	72	53	44	367	382	256	7-56 U/L
Aspartate transaminase (AST)	48	70	48	31	89	319	256	86	10-40 U/L
Total bilirubin	0.4	0.9	0.9	0.6	0.6	0.6	0.6	0.6	0.1-1.2 mg/dL
Direct bilirubin	0.3	N/A	N/A	N/A	N/A	N/A	N/A	N/A	0.0-0.3 mg/dL
Glucose	N/A	102	94	90	88	148	100	83	70-99 mg/dL (fasting)
White blood cells	9.6	6.6	7.1	6.1	6.2	13.4	10.6	9.5	4.5-11.0 × 10³/μL
Neutrophil	70.2	61.0	52.6	55.3	51.7	80.8	74.9	74.5	30%-75%
Lymphocyte	21.3	27.9	35.0	33.7	36.4	14.3	18.5	16.9	20%-45 %
Monocyte	7.6	8.8	9.6	8.6	9.8	4.4	5.7	7.6	0%-10%
Eosinophil	0.3	1.4	2.0	1.7	1.4	0.0	0.3	0.4	0%-6%
Basophils	0.3	0.6	0.7	0.5	0.5	0.1	0.4	0.3	0.5%-1%
Red blood cells	4.2	4.21	4.24	4.39	4.48	4.30	4.15	4.44	4.2-5.4 × 10^6^/µL (female)
Mean corpuscular volume (MCV)	83.6	84.1	84.0	82.7	82.8	81.6	82.2	82.7	80-96 fL (female)
Platelet	298	321	321	342	353	376	350	390	150-400 × 10³/μL
Hemoglobin	12.6	12.4	12.6	12.9	13.1	12.6	12.4	13.0	12.0-15.5 g/dL
Hematocrit	35.1	35.4	35.6	36.3	37.1	35.1	34.1	36.7	36.0%-44.0%
Urinalysis									
Urine pH	6.0	N/A	N/A	N/A	N/A	N/A	N/A	N/A	5-9
Protein	Negative	N/A	N/A	N/A	N/A	N/A	N/A	N/A	<30 mg/dL
Nitrite	Negative	N/A	N/A	N/A	N/A	N/A	N/A	N/A	Negative
Leukocyte esterase	Trace	N/A	N/A	N/A	N/A	N/A	N/A	N/A	Negative
Blood	Negative	N/A	N/A	N/A	N/A	N/A	N/A	N/A	Negative
Ketones	Negative	N/A	N/A	N/A	N/A	N/A	N/A	N/A	Negative

**Figure 1:**
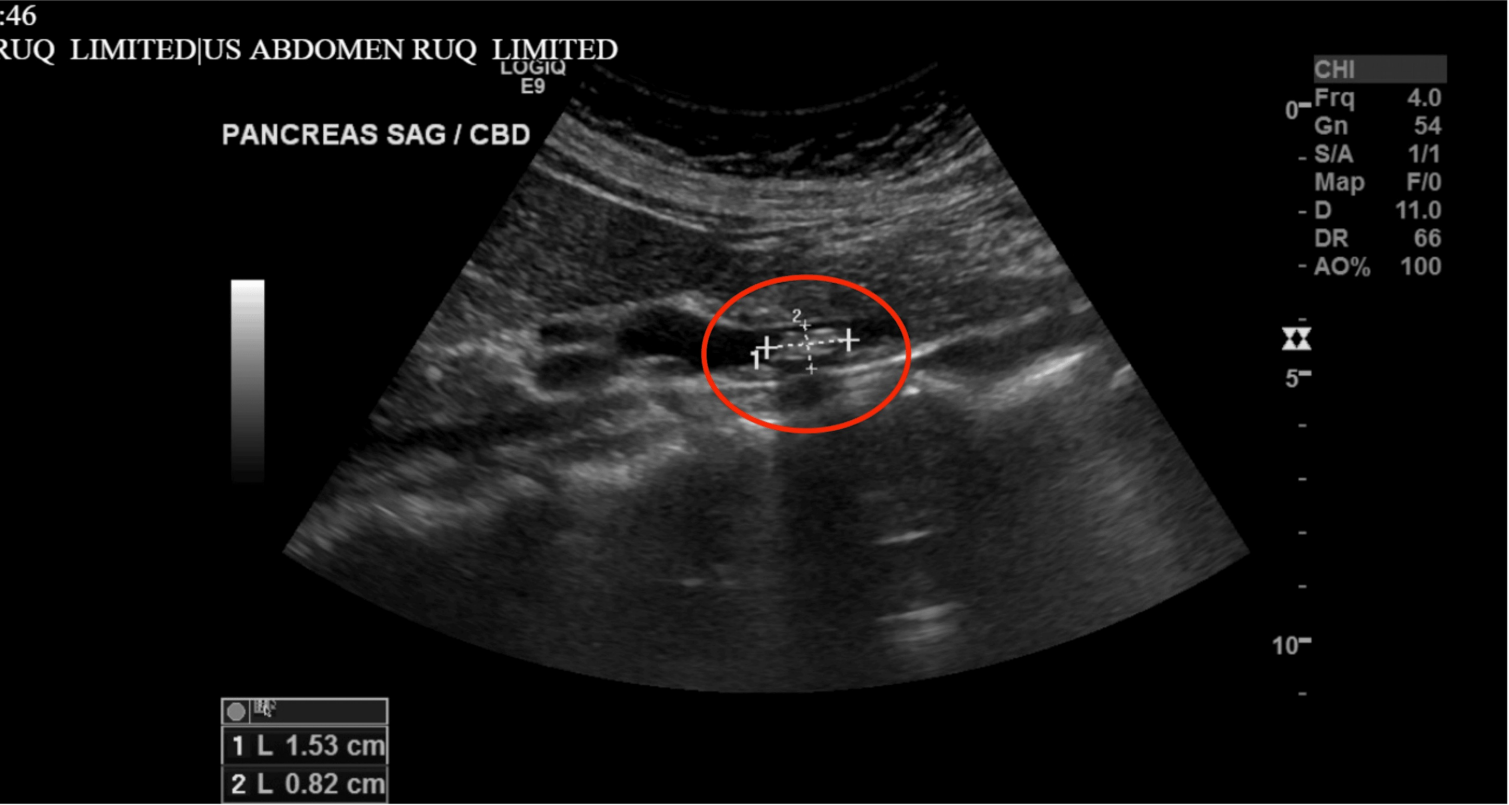
Initial right upper quadrant (RUQ) ultrasound showing a 1.5 cm x 0.8 cm stone (arrow) in the common bile duct (CBD), consistent with choledocholithiasis, but there were no sonographic findings of acute cholecystitis.

**Figure 2:**
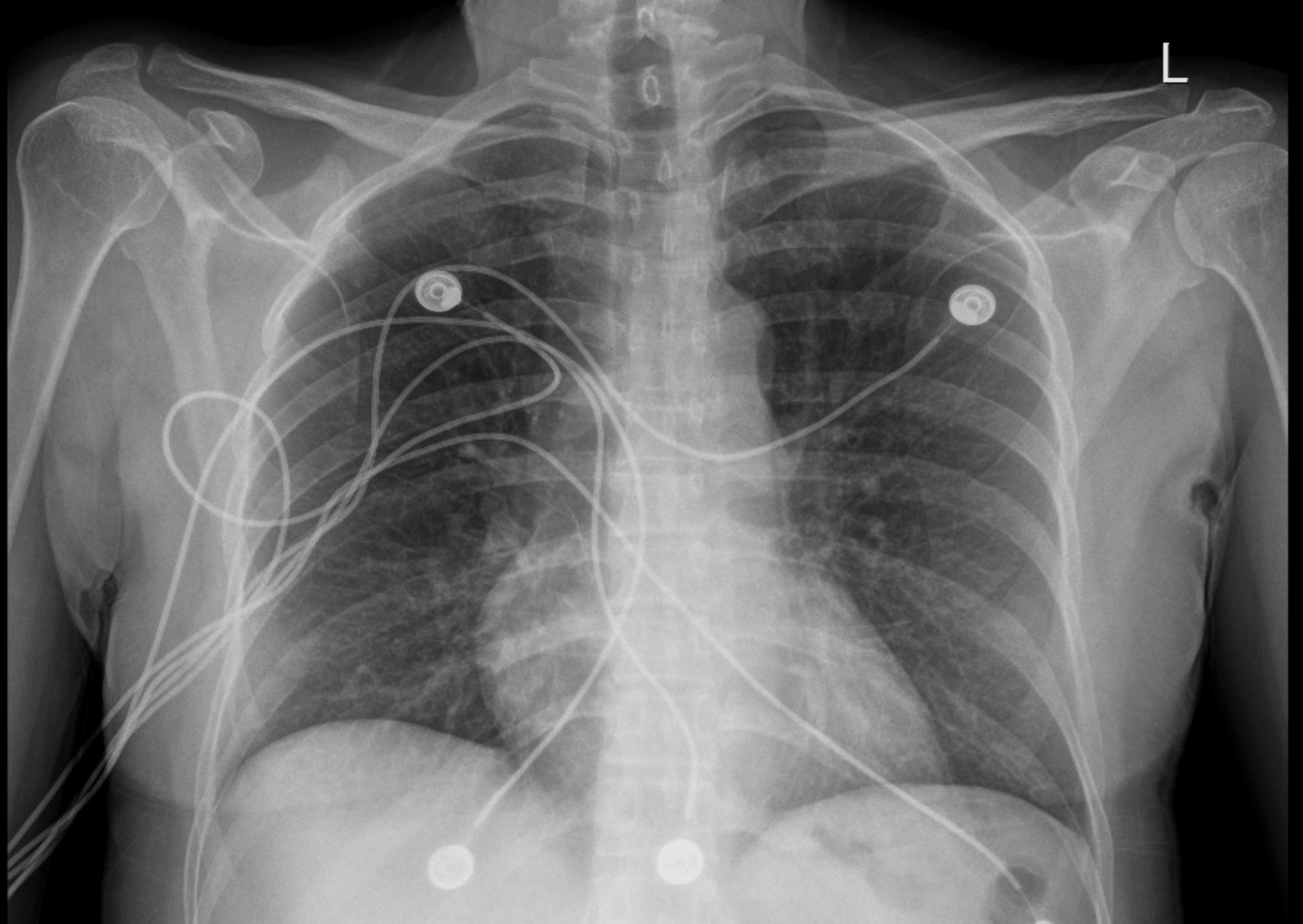
Chest X-ray on admission. No focal pulmonary consolidations, pleural effusions, or pneumothorax are observed. The cardiomediastinal silhouette is normal in size and appearance.

**Figure 3:**
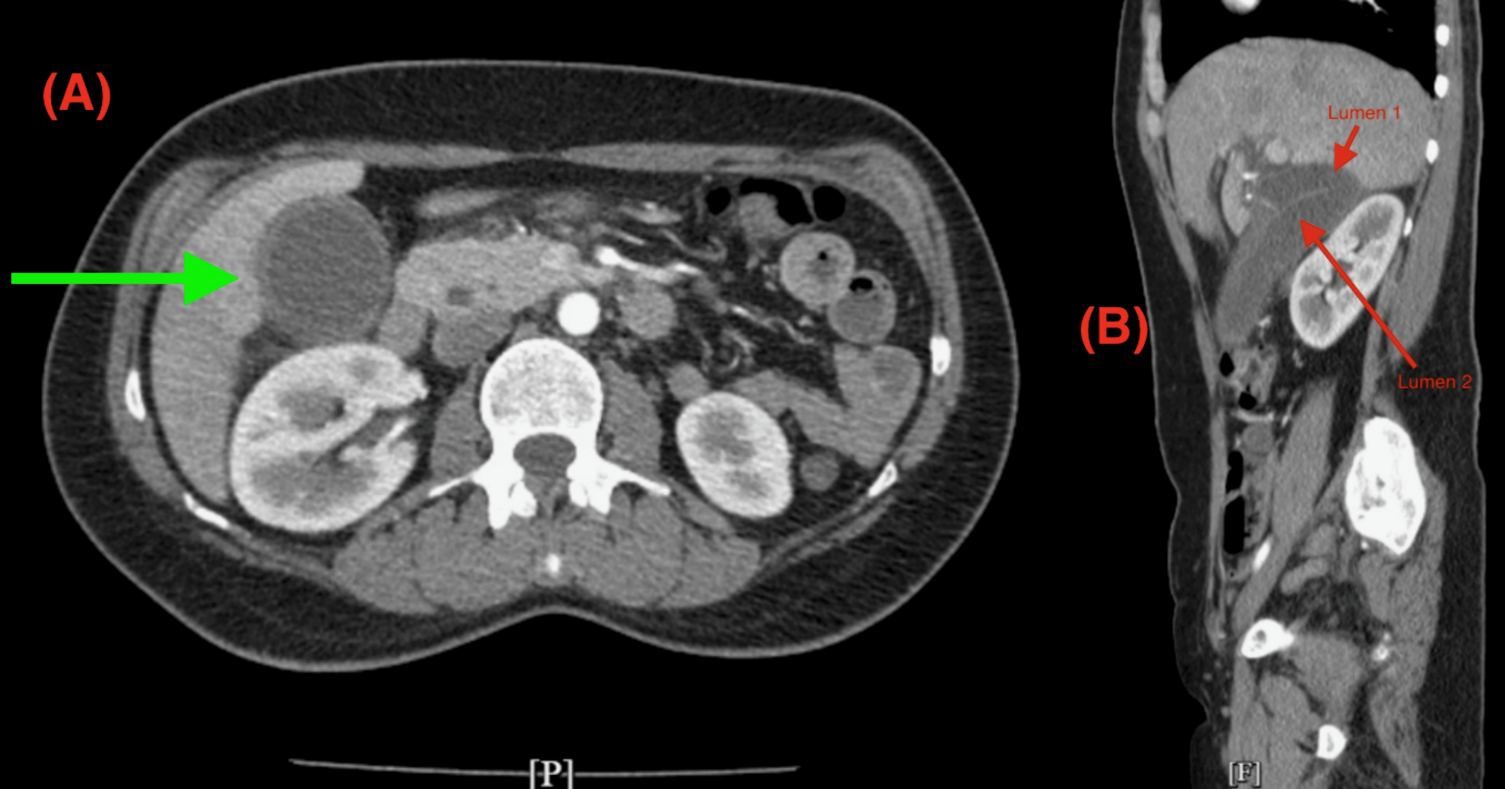
Abdominal computed tomography (CT). (A) Transverse cross-section showing a thickened gallbladder wall (green arrow), indicative of acute cholecystitis. (B) Sagittal cross-section demonstrating a Phrygian cap gallbladder with two lumens (red arrows).

**Figure 4:**
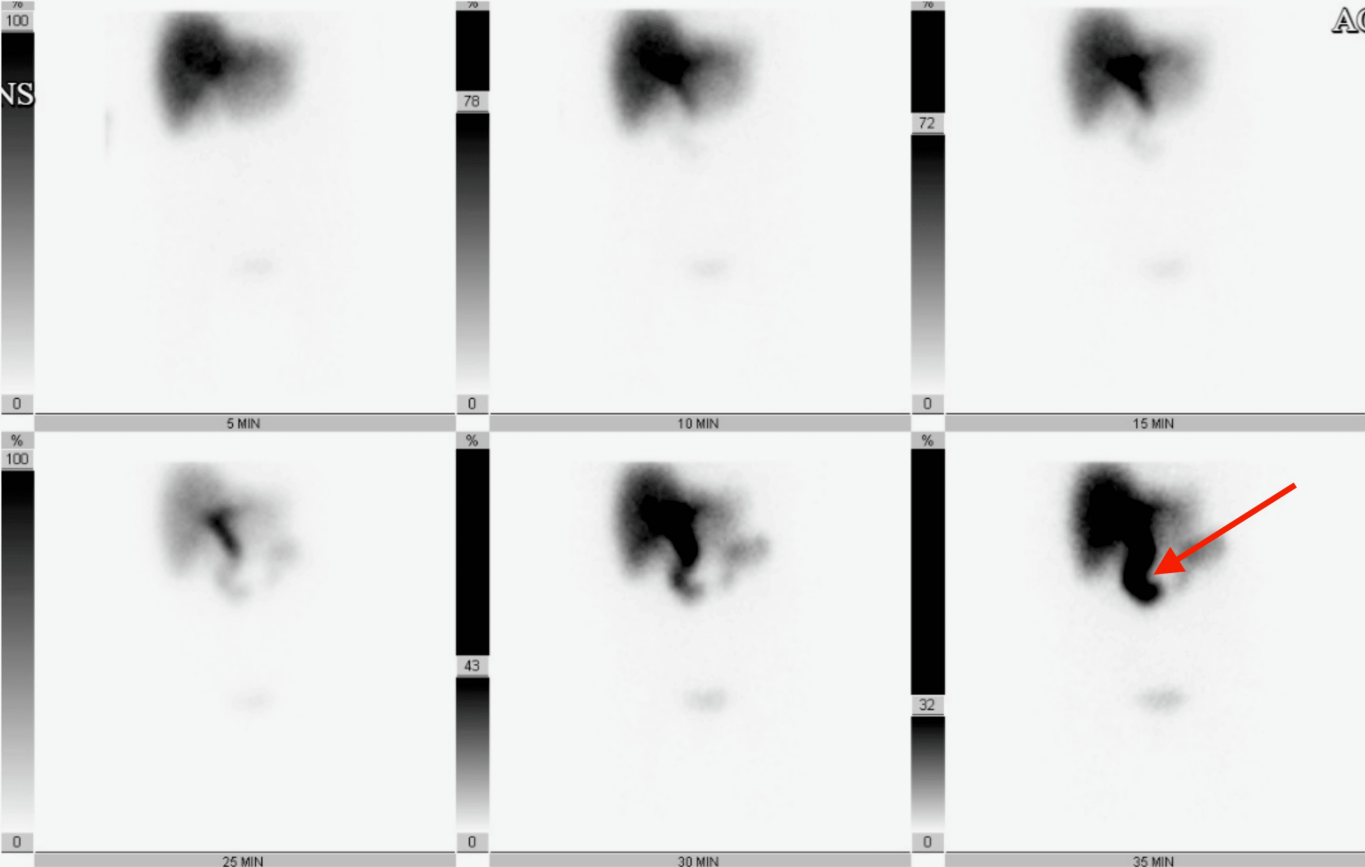
Hepatobiliary iminodiacetic acid (HIDA) scan. The patient was injected intravenously with 5 mCi Technetium-99m Choletec. Scintigraphy of the upper abdomen was performed sequentially for 60 minutes. There is activity in the common bile duct for 15 minutes. No radiotracer activity is visualized in the gallbladder within the first hour. In the delayed phase, radiotracer is seen in the proximal small bowel. The HIDA scan demonstrates scintigraphic evidence of acute cholecystitis and cystic duct obstruction.

**Figure 5:**
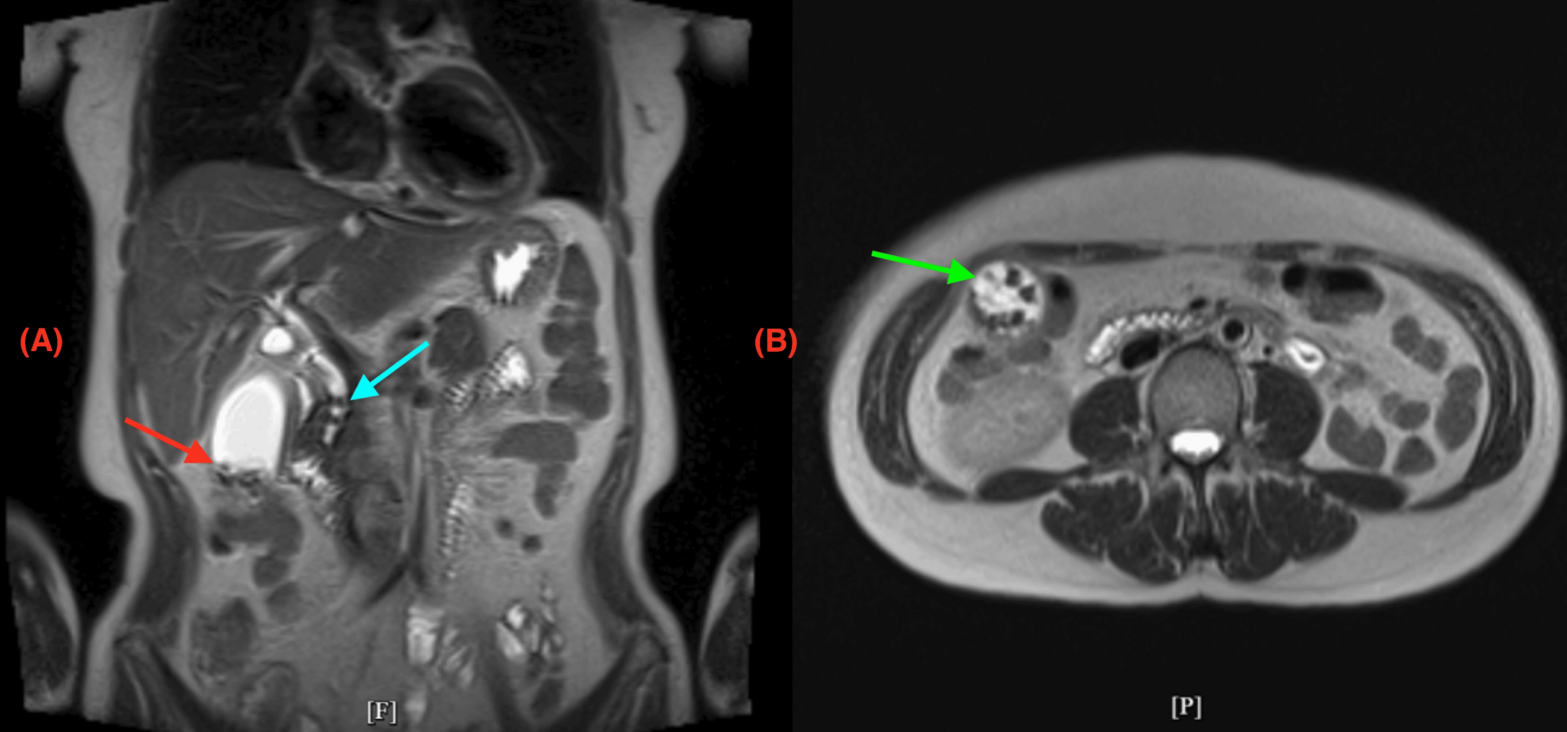
Magnetic resonance cholangiopancreatography (MRCP) imaging. (A) Coronal section demonstrating features suggestive of acute calculous cholecystitis. The common bile duct contains two filling defects, indicative of choledocholithiasis (blue arrow). The gallbladder appears distended and edematous, filled with multiple cholelithiases (red arrow), with adjacent trace-free fluid consistent with acute cholecystitis. No intrahepatic biliary ductal dilatation is observed. (B) Transverse section at the level of the gallbladder fundus showing multiple filling defects consistent with choledocholithiasis (green arrow).

**Figure 6:**
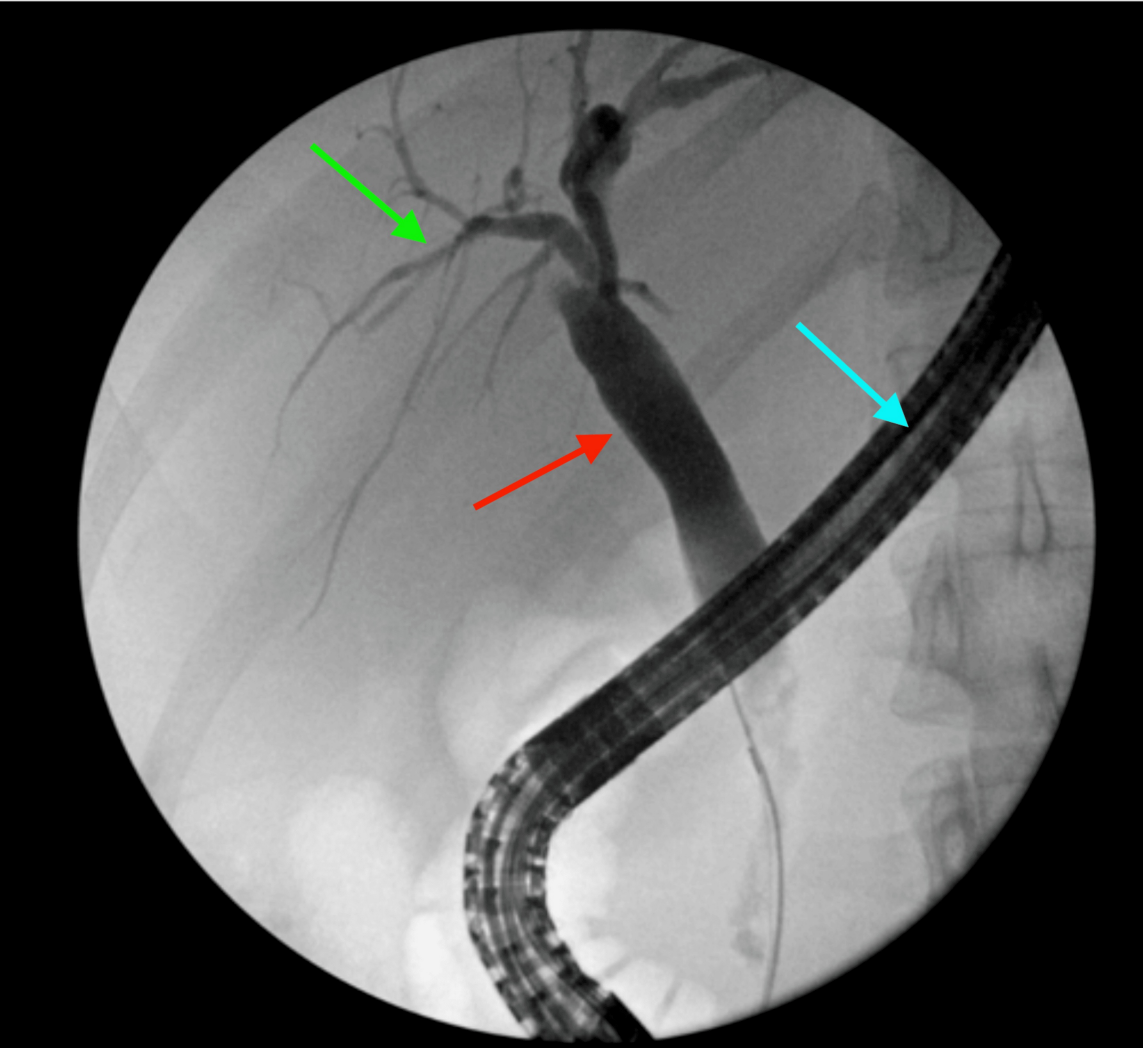
Endoscopic retrograde cholangiopancreatography (ERCP) showing the endoscope (blue arrow), biliary tree (green arrow), and dilated biliary duct (red arrow).

The folded fundus can obscure small gallstones or polyps on imaging, leading to a missed diagnosis. This is supported by the initial ultrasound of the abdomen which showed a 1.5 x 0.8 cm stone in the common bile duct, consistent with choledocholithiasis with no sonographic findings of acute cholecystitis (Figure 1). Chest X-ray (CXR) was unremarkable (Figure 2). Subsequent evaluation with an abdominal CT showed evidence of acute cholecystitis with common bile duct dilatation (11 mm) without evidence of radiopaque choledocholithiasis or pancreatic lesion (Figures 3a, 3b). Contradictory findings between the abdominal ultrasound and the CT led to further investigation with a HIDA scan. The HIDA scan showed evidence of acute cholecystitis with cystic duct obstruction (Figure 4). Cystic duct obstruction prompted an MRCP, confirming features suggestive of acute cholecystitis and a common bile duct filling defect, consistent with choledocholithiasis (Figure 5). ERCP successfully removed the stone from the common bile duct (Figure 6). A same-day laparoscopic cholecystectomy was then performed due to the diagnosis of acute cholecystitis. Postoperatively, the patient reported mild epigastric pain, but her vital signs remained stable and was afebrile. Serosanguinous drainage (20 cc) was noted from a drain in the RUQ, and her lipase (1440 U/L) and amylase (366 U/L) levels were elevated, likely secondary to the ERCP, but were trending downward across the hospital course (Table 1).

On days 5-7, the patient’s vitals remained stable, epigastric pain continued to subside, and drainage, amylase (100 U/L), and lipase (176 U/L) levels continued a downward trend. On day 6, the patient resumed normal bowel movements and was tolerating a low-fat diet well. The drain was removed, and the patient had no signs of fever, chills, or abdominal pain. On day 7, the patient was discharged home and was prescribed acetaminophen 650 mg every six hours as needed for pain and fevers for one week, and omeprazole 20 mg by mouth once a day, with instructions to follow up with her primary care provider within three days and with a general surgeon within 14 days. The patient was also advised to avoid heavy lifting for three weeks. The patient was instructed to seek medical attention if symptoms worsened.

## Discussion

The Phrygian cap, a congenital gallbladder variant, can mimic pathological conditions on imaging, complicating the diagnosis of acute cholecystitis, as seen in this case [7]. While typically considered a benign incidental finding, this anatomical variation can present diagnostic challenges, particularly in the context of acute cholecystitis with choledocholithiasis. There are two types of this variant: the retroserosal or concealed type, in which the mucosal fold projects into the lumen and is not externally visible, and the serosal or visible type, where the peritoneum follows the bend in the fundus, reflects on itself, and is externally visible [8].

In this case, the Phrygian cap created a sonographic challenge at first in assessing gallbladder wall integrity and pericholecystic fluid, necessitating further imaging for the diagnosis of acute cholecystitis. The initial ultrasound revealed gallstones without signs of acute cholecystitis; however, the patient's acute clinical presentation warranted further diagnostic evaluation. CT of the abdomen and pelvis revealed a distended, folded gallbladder with wall thickening, pericholecystic fluid, and mild fat stranding - findings consistent with acute cholecystitis. However, the CT did not show gallstones. The conflicting findings between the ultrasound and CT, along with the Phrygian cap, raised concerns about the ultrasound's accuracy, as the Phrygian cap can mimic gallstones [1,5].

Given this uncertainty, confirming the presence of gallstones was crucial before determining a treatment strategy. MRCP was performed before ERCP, as ERCP is more invasive and carries greater risks [9]. MRCP confirmed acute calculous cholecystitis with common bile duct filling defects consistent with choledocholithiasis. HIDA was then performed to assess cystic duct patency, which was found to be obstructed. The Phrygian cap necessitated the additional step of MRCP to confirm pathology before proceeding with the more invasive ERCP. Laparoscopic cholecystectomy (LC) was then conducted on the same day. A 2022 meta-analysis found that LC performed on the same day or within 72 hours was associated with a shorter length of hospital stay, shorter operative time, and lower risk of complications [10]. 

Despite ultrasound being the basis for diagnosing acute cholecystitis, additional imaging was needed due to diagnostic uncertainty. Complications were assessed with CT, cystic duct obstruction was confirmed by HIDA scan, and MRCP was performed to evaluate potential biliary involvement. All modalities provided distinct and clinically relevant information, guiding appropriate management.

The differential diagnosis for acute cholecystitis with a Phrygian cap includes acalculous cholecystitis, gallbladder torsion, choledocholithiasis, and cholangitis. Acalculous cholecystitis and gallbladder torsion were ruled out through imaging, specifically MRCP, ultrasound, and CT. Choledocholithiasis was confirmed with imaging. The patient’s white blood cell count remained within normal limits throughout her hospital stay, with a slight elevation on the day of the ERCP. Labs were drawn after the procedure, and the elevation was attributed to the inflammation caused by the ERCP, so cholangitis was not suspected.

Curci et al. and Santos et al. have reported cases of acute cholecystitis associated with a Phrygian cap, but there is limited literature beyond these reports [8,11]. In Curci et al., the primary challenge was determining whether pancreatitis resulted from gallstones or if the anatomical variation played a contributory role [11]. This case was similar to our case in that CT was not sufficient enough for a conclusive diagnosis, rather the addition of MRCP with three-dimensional reformatting made the diagnosis of acute biliary pancreatitis in the presence of a Phrygian cap gallbladder [11]. A similar challenge was found in Santos et al. in that abdominal US did not accurately reflect the true pathology [8]. On the US, they found a thickened gallbladder fold and what appeared to be two gallbladder polyps. However, after cholecystectomy, the polyps were found to be gallstones, and findings were then deemed consistent with chronic calculous cholecystitis [8]. These misinterpretations may be a result of a lack of published literature on similar findings. Our case emphasizes the importance of recognizing anatomical variations as potential confounders in biliary pathology, highlighting the need for comprehensive imaging. Notably, anatomical variation directly influenced the diagnostic process by necessitating sequential imaging to localize the gallstones.

Anatomical variations such as a Phrygian cap can significantly complicate clinical diagnosis and present unique challenges that require careful consideration and tailored approaches to ensure optimal patient outcomes. The Phrygian cap anomaly, as illustrated in this case, serves as a prime example of how such variations can impact medical practice. Its presence not only alters the typical anatomical landscape but also necessitates a heightened level of precision and adaptability during diagnostic imaging. This underscores the importance of recognizing and understanding rare anatomical deviations, as they can profoundly influence diagnostic strategies and patient care.

## Conclusions

This case highlights the importance of recognizing anatomical variations, such as a Phrygian cap when evaluating patients with suspected acute cholecystitis. The presence of a folded gallbladder can complicate imaging interpretation, potentially delaying diagnosis and management. In this case, multimodal imaging, including ultrasound, CT, MRCP, and HIDA, played a crucial role in accurately identifying both the anatomical variation and the underlying pathology. Timely intervention with ERCP and laparoscopic cholecystectomy led to a successful outcome, emphasizing the need for a thorough diagnostic approach in similar clinical scenarios.

## References

[1] Gallaher JR, Charles A (2022). Acute cholecystitis: a review. JAMA.

[2] Navuluri R, Hoyer M, Osman M, Fergus J (2020). Emergent treatment of acute cholangitis and acute cholecystitis. Semin Intervent Radiol.

[3] Meilstrup JW, Hopper KD, Thieme GA (1991). Imaging of gallbladder variants. AJR Am J Roentgenol.

[4] Arroyo CA (2014). Hepatobiliary. Pediatric Emergency Critical Care and Ultrasound.

[5] Gutman H, Landau O, Deutsch AA, Haddad M, Reiss R (1992). Acute acalculous cholecystitis versus acute calculous cholecystitis: review 1970-1988. Digestive Surgery.

[6] Walter K (2022). Acute cholecystitis. JAMA.

[7] van Kamp MJ, Bouman DE, Steenvoorde P, Klaase JM (2013). A phrygian cap. Case Rep Gastroenterol.

[8] Santos DMV, Santos DMAL, Branco A, Costa DJ (2016). Unsuspected Phrygian cap gallbladder in a 71 year old man. Brasília Médica.

[9] Kumar A, Mohanty NR, Mohanty M, Dash S (2023). Comparison of MRCP and ERCP in the evaluation of common bile duct and pancreatic duct pathologies. Front Med Technol.

[10] Poprom N, Suragul W, Muangkaew P, Vassanasiri W, Rungsakulkij N, Mingphruedhi S, Tangtawee P (2023). Timing of laparoscopic cholecystectomy after endoscopic retrograde cholangiopancreatography in cholelithiasis patients: a systematic review and meta-analysis. Ann Hepatobiliary Pancreat Surg.

[11] Curci FP, Cianci P, Montagna M, Cappiello M, Cafagna L, Restini E (2024). A 42-year-old woman with recurrent pancreatitis associated with gallstones and a Phrygian cap gallbladder. Am J Case Rep.

